# Association between becoming a carer in later life and changes in the trajectory of cognitive function: results from the English longitudinal study of ageing

**DOI:** 10.1093/ageing/afag132

**Published:** 2026-05-12

**Authors:** Baowen Xue, Anne McMunn, Rebecca Lacey, Nicola Brimblecombe, Martin Knapp, Magdalena Walbaum, Yuntao Chen

**Affiliations:** Research Department of Epidemiology and Public Health, University College London, 1-19 Torrington Place, London WC1E 7HB, London, UK; Research Department of Epidemiology and Public Health, University College London, 1-19 Torrington Place, London WC1E 7HB, London, UK; School of Health and Medical Sciences, City St George’s, University of London, London SW17 0RE, London, UK; Care Policy and Evaluation Centre, The London School of Economics and Political Science, London WC2A 2AE, London, UK; Care Policy and Evaluation Centre, The London School of Economics and Political Science, London WC2A 2AE, London, UK; Care Policy and Evaluation Centre, The London School of Economics and Political Science, London WC2A 2AE, London, UK; Division of Psychiatry, University College London, London W1T 7NF, London, UK

**Keywords:** unpaid care, cognition, PSM, older people

## Abstract

The influence of becoming a carer on cognitive function presents a complex picture. Variations in intensity, recipients and locations of caring may influence cognition differently. Using waves 2 to 10 (2004/2005 to 2021/2023) of the English Longitudinal Study of Ageing, we examined cognitive changes before and after transitioning into caring and explored whether these effects depend on care characteristics. We integrated the strengths of both ‘matching’ and ‘before and after’ methods. Using propensity score matching, we paired 2765 carers aged 50+ with 2765 non-carers. Using piecewise growth curve modelling, we modelled the changes in cognitive trajectories—memory and executive function—before and after transitioning into caring. Analyses also examined variations by care hours, location, recipient, number of people caring for, duration, sex and wealth. Carers were on average 60 years old, and 56% were women. We found that transition into lower-intensity caring responsibilities (5–9 hours/week), caring outside the household and caring for parents/parents-in-law exhibited a slower decline in executive function than non-carers. Those providing very intensive care (50+ hours/week), caring within the household or caring for a spouse/partner showed a more rapid decline. Memory changes followed a similar but much weaker pattern than for executive function. No evidence was found that sex or wealth moderated these effects. This suggests that the influence of caring on cognitive function is likely to be shaped by care-related characteristics. Our findings underscore the importance of preventing carer overload. While caring may help preserve cognitive function, excessive caring demands appear to accelerate cognitive decline.

## Key Points

- Lower-intensity, non-household and parental care were linked to slower executive decline.
- Those providing very intensive care, caring within the household or caring for a spouse/partner showed a more rapid decline.
- No evidence was found that sex or wealth moderated these effects.

## Introduction

By 2040, about 20% of the adult population in England will be living with major illnesses [1]. Many of them will require care not only from professionals but also from family members and friends. The literature commonly defines carers as individuals who provide (usually) unpaid support to those with physical or mental health conditions or disabilities, typically within a social relationship, such as family or friends [2]. Understanding the health outcomes of carers is essential. The ‘use it or lose it’ theory suggests that the social and intellectual engagement involved in caring might help preserve cognitive abilities and slow cognitive decline [3]. However, the ‘stress process model’ [4] suggests that caring is often associated with poor mental health outcomes [5], which could, in turn, lead to cognitive impairment [6, 7]. The stress process framework also suggests that stress effects can be modified by the availability of resources. Caring is more likely to be appraised negatively when resources are limited, but positively when resources are adequate [8, 9]. This underscores the importance of considering socioeconomic resources and variations in caring intensity, recipient characteristics, location and duration.

We identified 14 longitudinal studies, published between 1 January 2000 and 1 October 2025, assessing the influence of caring on cognitive function (Details in Appendix 1). Findings were mixed, potentially due to the unclear role of care characteristics. One longitudinal study has explored the role of care hours, reporting a positive association between caring hours and cognitive functioning in low- and moderate-intensity carers, but not in high-intensity carers, among older Chinese adults [10]. No research has investigated the influence of care locations or duration on cognitive function. Existing studies have typically either compared carers with non-carers without accounting for cognitive changes before and after the transition into caring—potentially overlooking unmeasured confounding and health-related selection into caring roles—or focused solely on cognitive changes among carers, without a non-caring comparison group. Only one study has examined changes in cognitive function before and after individuals assumed caring responsibilities [11]. It found that transitioning into caring was associated with modest declines in cognitive function. However, the study was based on a relatively small sample—251 carers and an equal number of non-carers—and did not account for hours of care provided or other care settings.

There may be inequalities in the caring experience based on sex and socioeconomic status. Research indicates that women disproportionately provide personal care in later life and that long hours of caring are more common among disadvantaged households [12]. Sex and socioeconomic status are also well-established determinants of cognitive ageing in later life [13, 14]. However, it is less clear whether these inequalities extend to the relationship between becoming a carer and cognitive changes.

Using longitudinal data from the English Longitudinal Study of Ageing (ELSA), we aimed to (i) model the trajectories of cognitive function before and after individuals become carers, comparing these with matched non-carers; and (ii) examine whether the influence on cognitive function varies based on caring hours, location, duration, care recipient, number of people caring for, sex and socioeconomic status.

## Methods

### Study population

We utilised ELSA, a representative longitudinal study of individuals aged 50+, as well as their partners, residing in private households in England [15]. ELSA commenced in 2002/2003 (individual response rate 67%), followed by nine subsequent waves of biennial interviews. Since caring information was not collected in wave 1, we aggregated data from wave 2 (2004/2005) to wave 10 (2021/2023) and excluded those partners younger than 50 years old. Ethical approval for ELSA was obtained from the London Multicentre Research Ethics Committee, and all participants provided full, informed, written consent.

A total of 6090 participants aged 50+ were reported to be carers in at least one of the waves (i.e. ever carers). To assess the changes in cognitive function, we excluded those who were not interviewed at least once before and once after the transition into caring (e.g. carers who only participated in one wave or already carers when first entered the survey), resulting in a sample size of 3093. After excluding missing values for sociodemographic characteristics, a sample of 2767 carers remained. The carers and non-carers were then paired 1:1 on the propensity scores (see Statistical methods), and 2765 (99.9%) carers were successfully matched, and thus, 2765 carers and 2765 matched non-carers were used in the data analysis (Flow chart in Appendix 2).

### Measures

#### Care characteristics

Caring status was measured by ‘Did you look after anyone in the past week?’ Those who answered yes were considered to be carers. Carers were asked how many hours in the past week they had looked after someone (categorised as <5, 5–9, 10–19, 20–49 and 50+ hours/week) and whether the person they cared for lived with them. Carers were also asked how many people they looked after (categorised as 1, 2 and 3+) and their relationship to the person(s). Four binary variables were created: care for spouse/partner, care for parent/parent-in-law, care for other relative and care for friend/neighbour. People can contribute to more than one care relationship if they are caring for more than one person. We did not include child/grandchild care. Care characteristics were taken from the first time of transition into caring since wave 2. Care duration was calculated as the total number of waves of caring since wave 2.

#### Cognitive function

Executive function was measured using a word-finding task (semantic verbal fluency), a test of how quickly participants could name as many different animals as possible in 1 minute (ranging from 0 to 63). Executive function was measured at every wave except wave 6 [16, 17]. We used averaged score at wave 5 and wave 7 for wave 6 [17]. Memory was measured by word list learning. Participants were given 10 common words to remember, both immediately (immediate recall) and after a short delay (delayed recall). We combined the scores on immediate recall and delayed recall (ranging from 0 to 20), with higher scores indicating better memory [17]. To make them comparable, standard scores (z-scores) were used.

#### Characteristics used in matching

Characteristics included in the matching (see Statistical methods) were age, sex, ethnicity, educational qualification, employment hours combined with employment status, occupational social class, quartiles of household income, quartiles of wealth, partnership status, number of children under age 18 in the household, whether participants had physical impairments, ever had a stroke and elevated depressive symptoms. These covariates were taken from baseline (i.e. wave 2 or the first wave of participation in the survey). We additionally included the wave number when first participating in the survey (whether wave 2 or not) to account for different baseline time points. We also matched the total number of waves of participation to account for the difference in loss-to-follow-up between carers and non-carers (details in Appendix 3).

### Statistical method

#### Propensity score matching

Propensity score matching (PSM) was used when matching carers with non-carers. The propensity score serves as a balancing score, ensuring that, conditional on the propensity score, the distribution of observed covariates is balanced between carers and non-carers. Propensity scores were calculated using a logistic regression model based on covariates listed in the Measures section [18]. We used nearest-neighbour matching based on the propensity scores, with exact matching on age, sex, partnership status and wave first observed as these variables are key for becoming a carer.

#### Trajectory of cognitive function

After PSM, the trajectory of cognitive function was centred on the age of transition into care. Carers’ age of transition was applied to their matched non-carers. As there were 9 waves of data available, we were able to estimate trajectories of cognitive function up to 18 years before and after becoming a carer. However, due to limited data at the most distant time points (number of observations <50), we excluded those time points, and thus, we showed the trajectories of cognitive function up to 18 years before and 16 years after becoming a carer (i.e. year −18 to year 16 in the time scale).

To statistically test the changes in cognitive function before and after becoming a carer, we employed piecewise linear growth curve modelling (level 1: observations, level 2: individuals, level 3: households). The cognitive function trajectory was partitioned into 2 linear segments with the year at the transition point as the ‘knot’ (i.e. year 0), so that years −18 to 0 represent the years before the transition, and years 0 to 16 represent the years after the transition (Details in Appendix 4). We examined interactions between care status/care characteristics and slope changes of cognitive function (post-transition minus pre-transition) to determine whether the changes differed between carers and non-carers or by care characteristics [19].

We further adjusted for covariates (see Measures) in the piecewise modelling. This ‘doubly robust’ estimation combines the regression model with PSM, making sure that the effect estimator is robust even if there is a misspecification of one of these models [20].

To visualise the results, we showed both the predicted piecewise linear trajectory of cognition outcomes (in the main text) and the predicted cognition at each year (in the appendices) before and after the transition, for carers and non-carers separately.

In the sensitivity analysis, we additionally adjusted for baseline cognitive function.

#### Sex and wealth differences

Using the piecewise modelling mentioned above, we tested differences in the association between becoming a carer and cognitive function by sex and wealth (continuous variable), using three-way interaction terms (care × slope change × sex; care × slope change × wealth).

In the sensitivity analysis, we excluded sex from the PSC when testing the sex difference in the association, and excluded wealth from the PSC when testing the wealth difference in the association.

## Results

### Care characteristics

Carers and matched non-carers showed very similar baseline characteristics (except for household income), suggesting an overall good balance after PSM (Appendix 5). Carers were on average 60 years old, and 56% were women. As shown in Table 1, 33% of carers cared for less than 5 hours/week, but one in five carers cared for 50+ hours/week. Most carers were caring for one person (79%), caring outside the household (57%) and had provided care for 1 year (58%). Approximately 41% were caring for a spouse/partner, and 28% were caring for parents/parents-in-law. Women carers were slightly more likely to care for 5+ hours/week, for more than one person, for longer years and to care outside the household. Some of the care characteristics intersect (Appendix 6). Long hours of care per week are concentrated among individuals providing care within the household and those caring for a spouse. However, these intensive hours do not necessarily correspond to caring for multiple people or to longer overall care duration.

**Table 1:** Care characteristics of carers.

	Men carers (*N* = 1212)		Women carers (*N* = 1553)		Total (*N* = 2765)	
Care characteristics	*N*	%	*N*	%	*N*	%
**Weekly hours spent caring for older people**						
<5 hours	414	34	504	32	918	33
5–9 hours	210	17	288	19	498	18
10–19 hours	180	15	207	13	387	14
20–49 hours	145	12	205	13	350	13
50+ hours	263	22	349	22	612	22
**Number of people caring for**						
1	994	82	1194	77	2188	79
2	146	12	209	13	355	13
3+	72	6	150	10	222	8
**Care location**						
Inside household	606	50	594	38	1200	43
Outside household	606	50	959	62	1565	57
**Care recipient**						
Spouse/partner	578	48	540	35	1118	41
Parent/parent-in-law	303	25	464	30	767	28
Other relative	98	8	179	12	277	10
Friend	251	21	408	26	659	24
Other	26	2	33	2	59	2
**Care duration (year)**						
1	741	61	860	55	1601	58
2	278	23	361	23	639	23
3+	193	16	332	21	525	19

The % of care recipients does not sum to 100 as some carers are caring for multiple people. Full response categories were shown for information but were collapsed to care for spouse/partner, parent/parent-in-law and any other for analyses.

### Change in scores of cognitive function before and after becoming a carer

**Figure 1:**
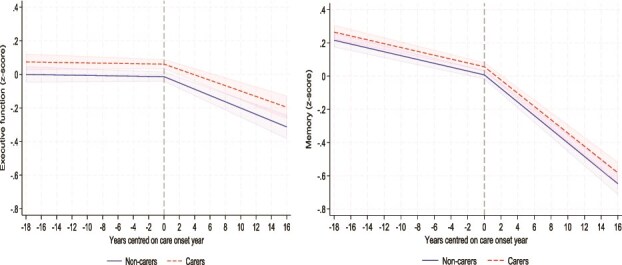
Predicted piecewise trajectories of executive function (left) and memory (right) with 95% confidence intervals before and after becoming a carer by care status, comparing carers and matched non-carers.

Piecewise models showed the relationship between becoming a carer and changes in scores of cognitive function. By just looking at care status (Figure 1), transitioning into a caring role was not associated with changes in either executive function scores or memory scores, as carers and non-carers showed similar changes in cognitive trajectory over time. Interaction results between care status and slope changes of cognitive function scores are shown in Appendix 7.

**Figure 2:**
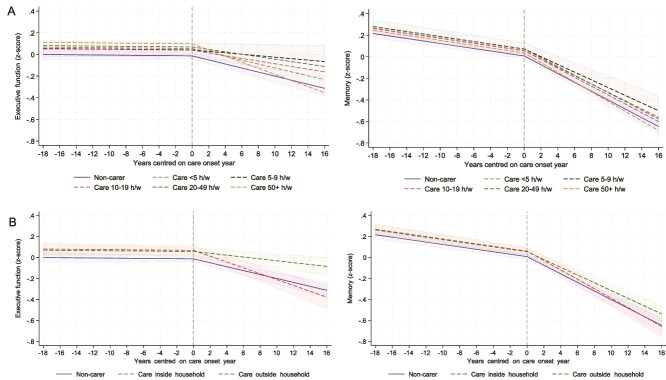
Predicted piecewise trajectories of executive function (left) and memory (right) with 95% confidence intervals before and after becoming a carer by care hours (panel A) and by care location (panel B), comparing carers and matched non-carers.

However, when looking at care characteristics, our results suggest a more complex picture. To start with caring hours, compared to non-carers (Figure 2A left), those caring for 5–9 hours/week showed slower decline in executive function scores (slope change = 0.012, *P* = .020), but those caring for 50+ hours/week showed faster decline in executive function scores (slope change = −0.010, *P* = .067, Appendix 7). No differences were found between those caring for 10–49 hours/week and non-carers (Appendix 7).

Looking at care location in Figure 2B left, those caring inside the household showed a faster decline in executive function scores than non-carers (slope change = −0.009, *P* = .025), while those caring outside the household showed a slower decline (slope change = 0.010, *P* = .005, Appendix 7) in executive function scores.

Memory changes followed a pattern similar to executive function, though much weaker, in relation to care hours and location (Figure 2 right), but many of these associations were not statistically significant.

**Figure 3:**
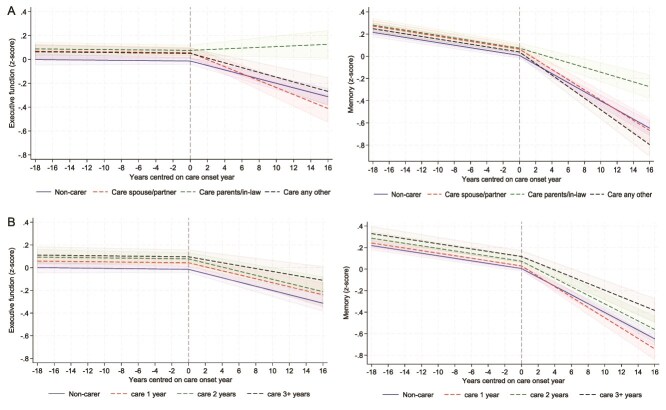
Predicted piecewise trajectories of executive function (left) and memory (right) with 95% confidence intervals before and after becoming a carer by care recipient (panel A) and by care duration (panel B), comparing carers and matched non-carers.

In terms of the care recipient (Figure 3A), caring for parents/parents-in-law showed an improvement in executive function (slope change = 0.022; *P* < .0001) and a slower decline in memory scores compared to non-carers (slope change = 0.019; *P* < .0001), but caring for a spouse/partner was associated with a faster decline in executive function scores (slope change = −0.011, *P* = .013, Appendix 7) than non-carers.

**Figure 4:**
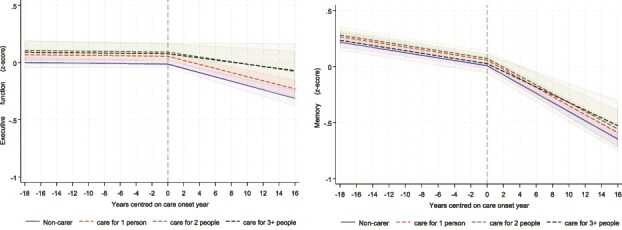
Predicted piecewise trajectories of executive function (left) and memory (right) with 95% confidence intervals before and after becoming a carer by number of people caring for, comparing carers and matched non-carers.

Regarding care duration (Figure 3B), only those caring for 3 + years showed a slightly slower decline in memory scores (coefficient = 0.010, *P* < .017, Appendix 7) than non-carers. Care duration was not associated with executive function scores. The number of people being cared for was associated with neither executive function nor memory scores (Figure 4).

Sensitivity analysis by adjusting for baseline cognitive function showed consistent results as the main analysis (Appendix 7, right).

The predicted cognitive function scores at each year before and after the transition by care status and care characteristics are shown in Appendix 8.

### Differences by sex and wealth

Results from three-way interaction showed that the association between caring status/caring characteristics and cognitive function scores did not differ by sex or wealth (Appendix 9). Sensitivity analysis by excluding sex and wealth in the PSM showed similar results that sex or wealth are not effect modifiers (results are not shown in tables).

## Discussion

We found that carers with lower-intensity responsibilities (5–9 hours/week), those caring outside the household and those caring for parents/parents-in-law exhibited a slower decline in executive function scores than non-carers. In contrast, those providing very intensive care (50+ hours/week), caring within the household or caring for a spouse/partner showed a more rapid decline than non-carers. Memory changes followed a similar but much weaker pattern. No evidence was found that sex or wealth moderated these effects.

The findings that becoming a less intensive carer is associated with a slower decline in executive function scores are in line with the ‘use it or lose it’ concept [3] and previous longitudinal studies [10, 21]. From this ‘use it or lose it’ perspective, taking on manageable levels of caregiving may provide cognitively stimulating activities and coordinating care that help maintain executive functioning in later life. It is likely that engaging in less intensive cognitive caregiving tasks, such as providing a few hours of support outside the household, may help carers maintain their cognitive health as they age. It is also possible that the caring role is associated with increased physical activity, which acts as a protective factor against cognitive decline [22, 23]. However, the protective effects suggested by the ‘use it or lose it’ framework appear to diminish—and even reverse—when caring becomes highly intensive. Carers providing 50+ hours of care/week exhibited accelerated cognitive decline, indicating that the cognitive stimulation associated with caring is overshadowed by the demands of high-intensity care. These individuals are typically full-time carers, leaving little opportunity for paid employment or social engagement, which are important for cognitive health. The intensity of such care may lead to feelings of loneliness and disrupt sleep, further compounding its negative effects on cognition [24, 25]. Similarly, carers who provide care inside the household or care for partners often face particularly demanding responsibilities and emotionally charged responsibilities, frequently involving extended hours with limited respite. Such care arrangements could also introduce financial strain if carers are forced to reduce their paid employment. These patterns align with the ‘stress process’ model [26], which emphasises that high and chronic exposure to stress may make carers more likely to experience poorer health outcomes than non-carers. Our findings support previous studies that transitioning to intensive care is linked to a more pronounced worsening of mental health [19]. The mental health challenges linked to caring may counterbalance the cognitive benefits of the social and intellectual engagement involved in caring activities.

Our results for memory showed similar patterns to those for executive function, though the associations were much weaker. As caring involves complex decision-making, problem-solving and communication, it may have a stronger connection to executive function than to memory [27].

We also found that those providing care for 3 or more years showed a slower rate of memory decline. This pattern suggests that longer-term carers may have established support systems for themselves and their care recipients, potentially allowing greater engagement in other activities and reducing overall strain. Alternatively, it is possible that individuals who provided care for less than 3 years discontinued caring due to emerging cognitive difficulties themselves. While our models accounted for health-related selection into caring, potential health-related attrition from caring may still influence these findings. No significant associations were observed between care duration and executive function. Taken together, the observed associations between care duration and cognitive outcomes should be interpreted with caution.

We observed no differences in the effect of becoming a carer on cognitive changes by sex. Although previous research suggests that care tasks are often more intense for women than for men, the sex differences in care hours diminish in later life [12]. Regarding wealth, carers in more advantaged households might have greater access to formal care and experience less financial stress. However, we found no differences in the effect of caring on cognitive changes based on wealth. This aligns with previous research, which also found no differences in the impact of caring on mental health by sex or household income [19]. From a public health perspective, though, this does not mean we can overlook inequalities in cognitive function among carers. We found that carers with higher wealth had higher cognitive function at baseline. Although the speed of cognitive decline during the transition into caring was similar across wealth levels, the initial cognitive disadvantage means that those from low-wealth households continued to have lower cognitive function throughout the observation period.

### Strengths and limitations

We used a representative longitudinal study of individuals aged 50+ in England and used ‘doubly robust’ estimation to minimise potential sociodemographic differences between carers and non-carers. We then assessed the trajectory of cognitive function changes, spanning several years both before and after taking on care roles. However, our study has some limitations. We did not have information on the specific health conditions of the care recipient. Care information was based on activities in the past week, which is sensitive to short-term fluctuations and may therefore introduce non-differential misclassification. Our dataset lacked information regarding the care history of individuals before their inclusion in the survey. Given that our findings relied on self-report data, it is possible that some individuals may not readily identify themselves as carers. Animal naming, which is consistently measured across ELSA waves, may capture only a subset of executive-function components rather than the full breadth of the construct. Furthermore, the potential non-participation or attrition of individuals with dementia or carers providing intensive care may introduce bias to our findings. We did not examine the impact of exiting a caring role, which is an important area for future research. Our analysis focused on the association between becoming a carer, care characteristics and changes in cognitive function. Future studies could explore the mechanisms linking different caring characteristics to cognitive changes, for example, through mediation analysis. Some care characteristics are interrelated. More complex composite profiles of caring are likely to emerge, and these will require larger samples of carers in future research.

## Conclusion

Providing less-intensive care was associated with a slower decline in cognitive function, but providing highly intensive care, inside household care or care for partners was associated with a faster decline in cognitive function. Our findings indicate that those intensive, in-household and spousal carers are most vulnerable to cognitive decline and may benefit from policies aimed at preventing carer overload, which could be achieved by enhancing access to and funding for formal care and longer-term ‘replacement care’ or other carer-focused support arrangements [28]. Lower-intensity carers, who may experience cognitive benefits, could be supported through measures that help them sustain manageable levels of engagement without escalating into high-intensity caring roles.

## Supplementary Material

afag132_aa-25-3371-File006

## Data Availability

ELSA data are available to download via the UK Data Service: https://ukdataservice.ac.uk/. Information on all data collected in the ELSA study is available on the study website: https://www.elsa-project.ac.uk/.
