# Supplementary material for: Association between becoming a carer in later life and changes in the trajectory of cognitive function: results from the English longitudinal study of ageing
Source: Age Ageing. 2026 May 12;55(5):afag132. doi: 10.1093/ageing/afag132 (PMC13167145; doi:10.1093/ageing/afag132)
Supplement: afag132_aa-25-3371-File006 [file afag132_aa-25-3371-file006.docx]

**Paper title: Association between becoming a carer in later life and changes in the trajectory of cognitive function: Results from the English Longitudinal Study of Ageing**

**Appendix 1** - Literature review of the association between care and cognitive function.

Search terms were: (caring [Title/Abstract]) OR (care* [Title/Abstract]) AND (cognition[Mesh]) AND ((longitudinal) OR (cohort)). We also hand-searched lists of references from relevant papers.

| **Author and year** | **Region, data and age** | **Objective** | **Exposure** | **Outcome** | **Model/analysis** | **Results** |
| --- | --- | --- | --- | --- | --- | --- |
| Lee et al. J Nerv Ment Dis. 2004[1] | Population-based study in the US: Nurses’ Health Study, women aged 70–79 years, | To investigate whether older women who provide care to their disabled or ill spouse have an increased risk of low cognitive function. | “Outside of your employment, do you provide regular care to a disabled or ill spouse” | 6 cognitive batteries  TICS (telephone version of MMSE) (8-41)  Delayed recall of 10-word list (0-10)  The East Boston Memory Test: immediate verbal recall (0-12) and delayed verbal recall (0-12)  Test of verbal fluency (0-38 animal named)  Digit Span Backwards (0-12)  Global score: z score | Logistic regression | Increased risks of low cognitive function on three of the cognitive tests among women who provided care to a disabled or ill spouse compared with women who did not provide any care. |
| Vitaliano et al. Psychology and Aging. 2005[2] | Participants were 110 spouse carers and their spouses  (care recipients with Alzheimer’s disease) and 105 noncarer spouses (and their spouses) over 2-year follow up | To examine relationships between caring and cognitive decline. | Spouse carers vs noncarer spouse | Shipley Institute of Living  Scale with two subset: vocabulary and general  reasoning. | ANOVA | Over 2 years, spouse carers declined by a small but  significant amount on Shipley Vocabulary. In  contrast, noncarer spouse did not change. |
| Vitaliano et al. Gerontologist. 2009[3] | Carers for spouses with Alzheimer's disease (n = 122) were compared with demographically similar noncarer spouses (n = 117) at study entry (Time 1 = T1), T2 (1 year later), and T3 (2 years after T1). | To examine relationships between caring and cognitive decline. | Spouse carers vs noncarer spouse | The DST was used to assess processing speed, complex attention psychomotor speed, cognitive–motor translation, and concentration. | Hierarchical linear modeling | Although carers started well below noncarers, they experienced a more rapid rate of decline than noncarers |
| Dassel et al. Gerontologist. 2017[4] | Population-based study in the US: Health and Retirement Study | To explore cognitive health outcomes between dementia and non-dementia caregivers. | Surviving spouses of those with and without a diagnosis of dementia | A composite measure of cognition based on three aspects of cognitive function: (a) episodic memory (10-item immediate and delayed recall test; range 0–20 points); (b) working memory (serial 7-s test; range 0–5 points); and (c) processing speed (backwards-counting; range 0–2 points). | Logistic regression | Spousal carers of persons with dementia experience accelerated cognitive decline themselves compared to nondementia carers. |
| Zwar et al.Soc Sci Med. 2018[5] | Population-based study in Germany: German Ageing Survey, aged 65+ | To investigate whether different informal caring types might influence cognitive functioning | Three caring types: (i) help around the house, (ii) looking after someone, (iii) performing nursing care services | Cognitive functioning was measured with a digit symbol test (DST), closely adapted from the Digit Symbol Substitution Test (DSST)-widely used as a global measure for cognitive function. | Fixed-effects regressions | Caring, in terms of looking after someone, can be beneficial for cognitive function, at least for female carers. Helping around the house and performing nursing care services are not associated with cognition. |
| Luo et al. Soc Sci Med. 2019[6] | Population-based study in China: The China Health and Retirement Survey (CHARLS), aged 50 and over, two years of follow-up time | To assess the effects of productive activities on cognitive decline over a two-year period. | Productive activities: caring for grandchildren, patients/parents in law, a spouse, informal helping, formal volunteering, and employment | Primary outcome: overall cognitive score (0-31).  Secondary outcome:  1. Episodic memory: a total score of immediate word recall and delayed word recall (0-20);  2. A total score of other cognitive tests including orientation, visuoconstruction, and numeric ability (0-11). | Generalized estimated equation | Caring for grandchildren, caring for a spouse, informal helping and formal volunteering are associated with reduced risk of cognitive decline over a two-year period. Caring for grandchildren and volunteering are most beneficial for urban women, informal helping is most beneficial for urban men, and paid employment is most beneficial for rural men. |
| Yuan & [Grühn](https://pubmed.ncbi.nlm.nih.gov/?term=Gr%C3%BChn+D&cauthor_id=32840611), Gerontologist 2021[7] | Population-based study in England: ELSA wave 1 to 8, aged 40 and over, maximum follow-up time of 16 years | To investigate the concurrent, cumulative and lagged effects of caregiving on well-being and cognition | “Did you look after anyone in the last week?” was asked twice in ELSA. Carers were those who answered both questions with “Yes” | Two well-being measures—life satisfaction and quality of life—and three cognitive functioning measures—immediate recall, delayed recall, and verbal fluency | Latent growth curve models with age as predictor. One concurrent, two cumulative, and two lagged effects (if possible) as predictors for the outcome variables | Current caregiving (concurrent effect) was related to worse well-being and better delayed recall. Little robust cumulative effect was found on cognition and well-being. Caregiving was related to worse well-being and better memory functioning 2–4 years later |
| García-Castro et al. Aging Ment Health 2022[8] | Population-based study in England: English Longitudinal Study of Ageing (ELSA), aged 50 and over, maximum follow-up time of six years | To explore further how being a carer, and its duration, are associated with four different cognitive tasks that represent three different cognitive domains | Respondents were asked whether they gave any informal care to anyone in the last month | Different measures of cognitive performance were assessed in ELSA at wave 7 such as verbal memory (immediate and delayed recall), executive function (verbal fluency) and working memory (serial 7 subtraction tasks) | Hierarchical regression analysis | Being a carer was positively associated with immediate and delayed recall and verbal fluency but not with serial 7. |
| Henning et al. Psychol Aging 2023[9] | Population-based study in Germany: German Ageing Survey, aged 40-85 at baseline, maximum follow-up time of nine years | To investigate the longitudinal association of prosocial activity and cognitive performance | Prosocial activities: volunteering, grandparenting, and family care | Processing speed (the Digit Symbol Substitution Test) | Latent growth curve in a structural equation model framework | No longitudinal association between prosocial activity and cognitive performance. |
| Su, J Gerontol B Psychol Sci Soc Sci. 2023[10] | Population-based study in China: CHARLS wave 2011, 2013, and 2018, aged 45 and over, maximum follow-up time of seven years | To investigate the correlation between informal care and carers’ cognitive functioning | Family caring provided by respondents over the past year  1. adult child caregiving: parents/parents in law  2. grandparent caregiving  3. spouse caregiving | Total cognitive score (0-32)  1. Memory (0-20)  2. Executive function (0-8)  3. Orientation function (0-4) | Growth curve model | A positive association between caring and cognitive functioning. The positive association was only found in low and moderate-intensity carers but not in high-intensity carers. Grandparents, adult children, and multiple carers had a higher average cognition level at age 60 than non-carers, and adult child carers exhibited a significantly slower rate of decline in cognition across ages. Spousal carers showed no significant disparities with noncarers. |
| Bhattacharyya et al., Journal of Aging and Health 2023[11] | Population-based study in the US: Midlife in the United States (MIDUS) study. maximum follow-up time of ten years | To examine whether informal caregivers performed worse, better, or similar to non-caregivers on cognitive tests of executive functioning and episodic memory over 10 years. | Participants answered the question: “During the last 12 months have you, yourself, GIVEN personal care for a period of one month or more to a family member or friend because of a physical or mental condition, illness, or disability?” | Executive functioning and episodic memory | Multiple linear regression analyses | Caring may be associated with better episodic memory but not executive functioning over time among the middle-aged and older adults. |
| Elayoubi et al. Psychology and Aging 2023[12] | Reasons for Geographic and Racial Differences in Stroke (REGARDS) study in the US, with a maximum follow-up time of 14 years | To assess the role of caring in cognitive function and change in the US population. | Caring status was assessed at baseline by asking participants, “Are you  currently providing care on an on-going basis to a family member with a chronic illness or  disability?” | Global cognitive functioning, learning and memory, and executive functioning. | Propensity score matching for baseline carers and non-carers, then multilevel modelling was used to assess whether matched carers and non-carers  differed on baseline cognitive performance or change over time. | Results showed carers, compared to non-carers, had better baseline scores on global  cognitive functioning and word list learning, but caregiving status was not  associated with cognitive decline over time. |
| Guo et al. Reseach on Aging 2025[13] | Population-based study in China- China Health and Retirement Longitudinal Study (CHARLS), with a maximum follow-up time of seven years | This study investigates the longitudinal association between spousal caregiving types and cognitive trajectories among middle-aged and older Chinese adults | Spousal caregivers were defined as individuals whose partner had limitations in ADL or IADL, and indicated that the main helper was their spouse. | Cognitive function was measured by the Mini-Mental State Examination (MMSE). The range of cognition score is 0–31. | Latent growth curve model uses two latent factors: intercept (baseline level) and slope (rate of change). | Spouses who only provided IADLs care had slower cognitive decline compared to those who did not provide care, regardless of gender. However, those who provided ADLs caregiving had faster cognitive decline compared to non-caregivers, especially in women. |
| Elayoubi et al. J Gerontol B Psychol Sci Soc Sci.2025 [14] | Caregiving Transition Study in the US (251 carers and 251 non-carers)Caregiver and non-caregiver groups were individually matched on demographic and health history factors, including age, sex, race, education, marital status, self-rated health at baseline, and self-reported history of cardiovascular disease | To examine the longitudinal impact of caregiving transitions on cognition. | “Are you currently providing care on an ongoing basis to a family member with a chronic illness or disability?” | Global cognition, episodic memory, and verbal fluency, | Carers and non-carers were individually matched on demographic and health history factors.  Descriptive comparisons | Negative associations were found between caring transitions and cognition, but effects were domain-specific for memory and global cognition, and short-lived. |

.

**Appendix 2** – Flow chart of sample selection

**Appendix 3**

Age was measured in years. Ethnicity was measured as White and non-White. We were not able to distinguish more nuanced ethnic groups due to the small sample size. Educational qualifications were categorised into a university degree, lower than a university degree and no qualification. Employment status was combined with working hours, including working full-time, working part-time and not working. Occupational class was measured by the National Statistics Socio-economic Classification (NS-SEC) 3-class version (Higher managerial, administrative and professional occupations; Intermediate occupations, Routine and manual occupations, Never worked and long-term unemployed). Household income was measured by monthly total household net income divided by the OECD equivalence scale and was split into quartiles. Partnership status included married/cohabiting and neither. Wealth was measured by the net financial wealth of the household and was split into quartiles. Number of children under age 18 in the household was categorised as 0/1/2/3+. Physical impairment was measured as with ADL/IADL or not. Stroke was measured as ever had a stroke or not. Depressive symptoms were measured by the short version of CES-D scale, with a score of 3 or greater used to denote ‘caseness’.

**Appendix 4** – Piecewise linear growth curve modelling

In the piecewise linear growth curve modelling, cognitive function was the dependent variable. Independent variables include care status, segment 1, segment 2, and interaction term between care characteristics and segment 2. Coding of age centred on the first uptake of care, segment 1 and segment 2 are shown in the table below.

| **Age centred on the first uptake of care** | **Segment 1** | **Segment 2** |
| --- | --- | --- |
| -18 | 0 | 0 |
| -17 | 1 | 0 |
| -16 | 2 | 0 |
| -15 | 3 | 0 |
| -14 | 4 | 0 |
| -13 | 5 | 0 |
| -12 | 6 | 0 |
| -11 | 7 | 0 |
| -10 | 8 | 0 |
| -9 | 9 | 0 |
| -8 | 10 | 0 |
| -7 | 11 | 0 |
| -6 | 12 | 0 |
| -5 | 13 | 0 |
| -4 | 14 | 0 |
| -3 | 15 | 0 |
| -2 | 16 | 0 |
| -1 | 17 | 0 |
| 0 | 18 | 1 |
| 1 | 19 | 2 |
| 2 | 20 | 3 |
| 3 | 21 | 4 |
| 4 | 22 | 5 |
| 5 | 23 | 6 |
| 6 | 24 | 7 |
| 7 | 25 | 8 |
| 8 | 26 | 9 |
| 9 | 27 | 10 |
| 10 | 28 | 11 |
| 11 | 29 | 12 |
| 12 | 30 | 13 |
| 13 | 31 | 14 |
| 14 | 32 | 15 |
| 15 | 33 | 16 |
| 16 | 34 | 17 |

**Appendix 5** - Characteristics of carers and matched non-carers.

| **Baseline characteristics** | **Carers**  **(N=2765)** | **Matched non-carers (N=2765)** | **p value** |
| --- | --- | --- | --- |
| **Age (year), mean (SD)** | 60.3 (7.79) | 60.3 (7.79) | 1.00 |
| **Women (%)** | 56 | 56 | 1.00 |
| **Ethnicity (%)** |  |  | 0.21 |
| White | 96 | 97 |  |
| Non-white | 4 | 3 |  |
| **Partnership (%)** |  |  | 1.00 |
| Married/cohabiting | 83 | 83 |  |
| Neither | 17 | 17 |  |
| **Number of children under age 18 in household (%)** |  |  | 0.88 |
| **0** | 91 | 90 |  |
| 1 | 6 | 7 |  |
| 2 | 2 | 2 |  |
| 3+ | 1 | 1 |  |
| **Household income quartile (%)** |  |  | 0.002 |
| Lowest | 20 | 18 |  |
| 2 | 26 | 23 |  |
| 3 | 27 | 30 |  |
| Highest | 27 | 29 |  |
| **Wealth quartile (%)** |  |  | 0.15 |
| Lowest | 21 | 19 |  |
| 2 | 22 | 21 |  |
| 3 | 28 | 27 |  |
| Highest | 30 | 33 |  |
| **Education qualification (%)** |  |  | 0.76 |
| University degree | 20 | 19 |  |
| Lower than university degree | 57 | 58 |  |
| No qualification | 24 | 23 |  |
| **Employment status (%)** |  |  | 0.30 |
| Part time | 23 | 23 |  |
| Full time | 32 | 33 |  |
| Not working | 45 | 43 |  |
| **Occupational class (%)** |  |  | 0.70 |
| Higher managerial, administrative and professional occupations | 39 | 40 |  |
| Intermediate occupations | 25 | 23 |  |
| Routine and manual occupations | 36 | 36 |  |
| Never worked and long term unemployed | 1 | 1 |  |
| **Depressive symptoms (%)** |  |  | 0.57 |
| Caseness (score>=3) | 18 | 18 |  |
| **Ever had a stroke (%)** |  |  | 0.55 |
| Yes | 1 | 1 |  |
| **Physical impairment(%)** |  |  | 0.55 |
| Yes | 18 | 17 |  |
| **Baseline wave number** |  |  | 1.00 |
| Wave 2 | 61 | 61 |  |
| Not wave 2 | 39 | 39 |  |

**Appendix 6**– Interrelation between care characteristics.

|  | **Care location** | | **Number of people caring for** | | | **Caring duration** | | | **Care recipient** | | | | |
| --- | --- | --- | --- | --- | --- | --- | --- | --- | --- | --- | --- | --- | --- |
| **Care hours/ week** | *Care inside* | *Care outside* | *1* | *2* | *3+* | *1 y* | *2y* | *3+y* | *Spouse/*  *partner* | *Parent/*  *parent-in-law* | *Other relative* | *Friend* | *Other* |
| <5h (%) | 18.2 | 81.8 | 80.5 | 14.6 | 4.9 | 63.7 | 21.6 | 14.7 | 17.9 | 25.8 | 12.2 | 43.6 | 3.1 |
| 5-10h (%) | 28.3 | 71.7 | 75.1 | 16.3 | 8.6 | 59.2 | 24.5 | 16.3 | 27.5 | 37.8 | 14.9 | 23.7 | 1.6 |
| 10-19h (%) | 42.9 | 57.1 | 78.0 | 12.4 | 9.6 | 56.1 | 22.0 | 22.0 | 40.3 | 35.4 | 9.3 | 19.4 | 1.0 |
| 20-49h (%) | 49.7 | 50.3 | 70.9 | 12.3 | 16.9 | 52.0 | 26.0 | 22.0 | 45.1 | 35.4 | 10.0 | 11.7 | 3.1 |
| 50+h (%) | 90.2 | 9.8 | 85.8 | 8.0 | 6.2 | 52.6 | 23.4 | 24.0 | 82.4 | 13.2 | 3.3 | 4.1 | 1.3 |

**Appendix 7**– Results of interactions between care characteristics and slope change before and after the care transition (N=2765)*

|  |  | **Without adjusting for baseline cognition** | | | | **Sensitivity analysis: Adjusted** **for baseline cognition** | | | |
| --- | --- | --- | --- | --- | --- | --- | --- | --- | --- |
|  |  | **Coefficient** | ***p*** | **Lower 95% CI** | **Upper 95% CI** | **Coefficient** | ***p*** | **Lower 95% CI** | **Upper 95% CI** |
| ***Care status*** |  |  |  |  |  |  |  |  |  |
| **Executive function** | Care × slope change | 0.003 | 0.382 | -0.003 | 0.009 | 0.004 | 0.236 | -0.002 | 0.009 |
| **Memory** | Care × slope change | 0.001 | 0.715 | -0.005 | 0.007 | 0.002 | 0.512 | -0.004 | 0.008 |
| ***Care hours*** |  |  |  |  |  |  |  |  |  |
| **Executive function** | Care <5 h/w × slope change | 0.001 | 0.864 | -0.008 | 0.009 | 0.001 | 0.737 | -0.007 | 0.010 |
|  | Care 5-9 h/w × slope change | 0.012 | 0.020 | 0.002 | 0.022 | 0.015 | 0.003 | 0.005 | 0.024 |
|  | Care 10-19 h/w × slope change | 0.006 | 0.296 | -0.005 | 0.018 | 0.006 | 0.264 | -0.005 | 0.018 |
|  | Care 20-49 h/w × slope change | 0.007 | 0.232 | -0.005 | 0.020 | 0.008 | 0.196 | -0.004 | 0.020 |
|  | Care 50+ h/w × slope change | -0.010 | 0.067 | -0.020 | 0.001 | -0.010 | 0.051 | -0.020 | 0.0001 |
| **Memory** | Care <5 h/w × slope change | 0.003 | 0.545 | -0.006 | 0.011 | 0.003 | 0.454 | -0.005 | 0.011 |
|  | Care 5-9 h/w × slope change | 0.005 | 0.291 | -0.004 | 0.015 | 0.007 | 0.128 | -0.002 | 0.017 |
|  | Care 10-19 h/w × slope change | -0.0003 | 0.961 | -0.011 | 0.011 | 0.001 | 0.926 | -0.010 | 0.011 |
|  | Care 20-49 h/w × slope change | 0.001 | 0.938 | -0.011 | 0.012 | 0.001 | 0.800 | -0.010 | 0.013 |
|  | Care 50+ h/w × slope change | -0.004 | 0.408 | -0.014 | 0.006 | -0.004 | 0.386 | -0.014 | 0.005 |
| ***Care location*** |  |  |  |  |  |  |  |  |  |
| **Executive function** | Care inside household × slope change | -0.009 | 0.025 | -0.017 | -0.001 | -0.009 | 0.025 | -0.017 | -0.001 |
|  | Care outside household × slope change | 0.010 | 0.005 | 0.003 | 0.017 | 0.011 | 0.001 | 0.004 | 0.018 |
| **Memory** | Care inside household × slope change | -0.004 | 0.326 | -0.011 | 0.004 | -0.003 | 0.376 | -0.011 | 0.004 |
|  | Care outside household × slope change | 0.004 | 0.227 | -0.002 | 0.010 | 0.005 | 0.122 | -0.001 | 0.011 |
| ***Number of people caring for*** |  |  |  |  |  |  |  |  |  |
| **Executive function** | 1 | 0.001 | 0.786 | -0.006 | 0.007 |  |  |  |  |
|  | 2 | 0.008 | 0.171 | -0.004 | 0.020 |  |  |  |  |
|  | 3+ | 0.010 | 0.214 | -0.006 | 0.025 |  |  |  |  |
| **Memory** | 1 | 0.0004 | 0.899 | -0.006 | 0.006 |  |  |  |  |
|  | 2 | 0.002 | 0.779 | -0.009 | 0.013 |  |  |  |  |
|  | 3+ | 0.006 | 0.385 | -0.008 | 0.021 |  |  |  |  |
| ***Care recipient*‡** |  |  |  |  |  |  |  |  |  |
| **Executive function** | Care for spouse/partner × slope change |  |  |  |  |  |  |  |  |
|  | Yes | -0.011 | 0.013 | -0.019 | -0.002 | -0.010 | 0.013 | -0.018 | -0.002 |
|  | No | 0.009 | 0.006 | 0.003 | 0.016 | 0.010 | 0.002 | 0.004 | 0.017 |
|  | Care for parent/parent-in-law × slope change |  |  |  |  |  |  |  |  |
|  | Yes | 0.022 | <0.0001 | 0.014 | 0.030 | 0.022 | <0.0001 | 0.015 | 0.031 |
|  | No | -0.008 | 0.020 | -0.015 | -0.001 | -0.007 | 0.029 | -0.014 | -0.001 |
|  | Care for any other × slope change |  |  |  |  |  |  |  |  |
|  | Yes | -0.001 | 0.768 | -0.009 | 0.070 | 0.0003 | 0.936 | -0.008 | 0.008 |
|  | No | 0.005 | 0.163 | -0.002 | 0.012 | 0.005 | 0.115 | -0.001 | 0.012 |
| **Memory** | Care for spouse/partner × slope change |  |  |  |  |  |  |  |  |
|  | Yes | -0.005 | 0.231 | -0.013 | 0.003 | -0.005 | 0.258 | -0.012 | 0.003 |
|  | No | 0.004 | 0.214 | -0.002 | 0.010 | 0.005 | 0.113 | -0.001 | 0.011 |
|  | Care for parent/parent-in-law × slope change |  |  |  |  |  |  |  |  |
|  | Yes | 0.019 | <0.0001 | 0.011 | 0.027 | 0.021 | <0.0001 | 0.013 | 0.028 |
|  | No | -0.009 | 0.004 | -0.016 | -0.003 | -0.009 | 0.007 | -0.015 | -0.002 |
|  | Care for any other × slope change |  |  |  |  |  |  |  |  |
|  | Yes | -0.011 | 0.005 | -0.019 | -0.003 | -0.010 | 0.008 | -0.018 | -0.003 |
|  | No | 0.008 | 0.018 | 0.001 | 0.014 | 0.009 | 0.008 | 0.002 | 0.015 |
| ***Care duration*** |  |  |  |  |  |  |  |  |  |
| **Executive function** | Care for 1 year × slope change | 0.001 | 0.791 | -0.007 | 0.009 | 0.002 | 0.609 | -0.006 | 0.010 |
|  | Care for 2 year × slope change | 0.001 | 0.894 | -0.008 | 0.010 | 0.001 | 0.789 | -0.008 | 0.010 |
|  | Care for 3+ year × slope change | 0.006 | 0.176 | -0.003 | 0.014 | 0.007 | 0.078 | -0.001 | 0.015 |
| **Memory** | Care for 1 year × slope change | -0.007 | 0.057 | -0.015 | 0.002 | -0.007 | 0.069 | -0.014 | 0.001 |
|  | Care for 2 year × slope change | 0.001 | 0.794 | -0.007 | 0.010 | 0.002 | 0.648 | -0.007 | 0.011 |
|  | Care for 3+ year × slope change | 0.010 | 0.017 | 0.002 | 0.017 | 0.012 | 0.003 | 0.004 | 0.019 |

* Reference group is non-carers. Slope change is calculated as the slope after transition minus the slope before transition. Each care characteristic is tested in separate models. **‡** Each type of care recipient is tested in separate models as some carers are caring for multiple people.

**Appendix 8**- Predicted levels of cognitive function at each time point before and after becoming a carer by care status, comparing carers and matched non-carers

Fig S1- Predicted levels of executive function (left) and memory (right) at each time point with 95% confidence intervals before and after becoming a carer by care status, comparing carers and matched non-carers.

Fig S2- Predicted levels of executive function (left) and memory (right) at each time point before and after becoming a carer by care hours, comparing carers and matched non-carers. (95% confidence intervals are not shown as they overlap with each other and made the figures difficult to see).

Fig S3- Predicted levels of executive function (left) and memory (right) at each time point with 95% confidence intervals before and after becoming a carer by care location comparing carers and matched non-carers.

Fig S4- Predicted levels of executive function (left) and memory (right) at each time point with 95% confidence intervals before and after becoming a carer by number of people caring for, comparing carers and matched non-carers.

Fig S5- Predicted levels of executive function (left) and memory (right) at each time point with 95% confidence intervals before and after becoming a carer by care duration, comparing carers and matched non-carers.

Fig S6- Predicted levels of executive function (left) and memory (right) at each time point with 95% confidence intervals before and after becoming a carer by care recipient, comparing carers and matched non-carers.

**Appendix 9** - Results of 3-way interactions between care status, slope change (before and after the care transition) and inequality factors (sex, wealth).

|  |  | **Coefficient** | ***p*** | **Lower 95% CI** | **Upper 95% CI** |
| --- | --- | --- | --- | --- | --- |
|  | ***Sex difference*** |  |  |  |  |
| ***Care status*** |  |  |  |  |  |
| **Executive function** | Care × slope change × women | 0.003 | 0.770 | -0.010 | 0.014 |
| **Memory** | Care × slope change × women | 0.008 | 0.181 | -0.004 | 0.020 |
| ***Care location*** |  |  |  |  |  |
| **Executive function** | Care inside household × slope change × women | 0.008 | 0.309 | -0.008 | 0.024 |
|  | Care outside household × slope change × women | -0.008 | 0.277 | -0.022 | 0.006 |
| **Memory** | Care inside household × slope change × women | 0.010 | 0.183 | -0.005 | 0.026 |
|  | Care outside household × slope change × women | 0.004 | 0.524 | -0.009 | 0.018 |
| ***Care duration*** |  |  |  |  |  |
| **Executive function** | Care for 1 year × slope change × women | -0.002 | 0.774 | -0.018 | 0.014 |
|  | Care for 2 year × slope change × women | 0.005 | 0.622 | -0.014 | 0.023 |
|  | Care for 3+ year × slope change × women | 0.004 | 0.663 | -0.013 | 0.021 |
| **Memory** | Care for 1 year × slope change × women | 0.006 | 0.416 | -0.009 | 0.022 |
|  | Care for 2 year × slope change × women | 0.002 | 0.863 | -0.016 | 0.019 |
|  | Care for 3+ year × slope change × women | 0.013 | 0.131 | -0.004 | 0.029 |
| ***Care recipient*** |  |  |  |  |  |
| **Executive function** | Care for spouse/partner × slope change × women | 0.005 | 0.548 | -0.012 | 0.022 |
|  | Not care for spouse/partner × slope change × women | -0.005 | 0.446 | -0.019 | 0.008 |
|  | Care for parent/parent-in-law × slope change × women | 0.003 | 0.770 | -0.014 | 0.019 |
|  | Not care for parent/parent-in-law × slope change × women | -0.001 | 0.893 | -0.015 | 0.013 |
|  | Care for any other × slope change × women | -0.012 | 0.171 | -0.030 | 0.005 |
|  | Not care for any other × slope change × women | 0.010 | 0.160 | -0.004 | 0.023 |
| **Memory** | Care for spouse/partner × slope change × women | 0.005 | 0.541 | -0.011 | 0.021 |
|  | Not care for spouse/partner × slope change × women | 0.008 | 0.264 | -0.006 | 0.021 |
|  | Care for parent/parent-in-law × slope change × women | 0.014 | 0.089 | -0.002 | 0.030 |
|  | Not care for parent/parent-in-law × slope change × women | 0.002 | 0.708 | -0.011 | 0.016 |
|  | Care for any other × slope change × women | 0.005 | 0.585 | -0.012 | 0.021 |
|  | Not care for any other × slope change × women | 0.014 | 0.039 | 0.001 | 0.027 |
|  | ***Wealth difference*** |  |  |  |  |
| ***Care status*** |  |  |  |  |  |
| **Executive function** | Care × slope change × wealth | -0.002 | 0.541 | -0.009 | 0.005 |
| **Memory** | Care × slope change × wealth | -0.001 | 0.778 | -0.007 | 0.005 |
| ***Care location*** |  |  |  |  |  |
| **Executive function** | Care inside household × slope change × wealth | -0.002 | 0.541 | -0.009 | 0.005 |
|  | Care outside household × slope change × wealth | -0.001 | 0.778 | -0.007 | 0.005 |
| **Memory** | Care inside household × slope change × wealth | 0.004 | 0.218 | -0.003 | 0.011 |
|  | Care outside household × slope change × wealth | 0.002 | 0.568 | -0.004 | 0.008 |
| ***Care duration*** |  |  |  |  |  |
| **Executive function** | Care for 1 year × slope change × wealth | 0.001 | 0.759 | -0.006 | 0.008 |
|  | Care for 2 year × slope change × wealth | -0.008 | 0.069 | -0.016 | 0.001 |
|  | Care for 3+ year × slope change × wealth | 0.003 | 0.443 | -0.005 | 0.010 |
| **Memory** | Care for 1 year × slope change × wealth | 0.005 | 0.153 | -0.002 | 0.012 |
|  | Care for 2 year × slope change × wealth | -0.004 | 0.333 | -0.012 | 0.004 |
|  | Care for 3+ year × slope change × wealth | 0.006 | 0.072 | -0.001 | 0.014 |
| ***Care recipient*** |  |  |  |  |  |
| **Executive function** |  |  |  |  |  |
|  | Care for spouse/partner × slope change × wealth | -0.004 | 0.284 | -0.012 | 0.003 |
|  | Not care for spouse/partner × slope change × wealth | <0.0001 | 0.932 | -0.006 | 0.006 |
|  | Care for parent/parent-in-law × slope change × wealth | 0.002 | 0.514 | -0.005 | 0.010 |
|  | Not care for parent/parent-in-law × slope change × wealth | -0.002 | 0.571 | -0.008 | 0.004 |
|  | Care for any other × slope change × wealth | -0.001 | 0.710 | -0.009 | 0.006 |
|  | Not care for any other × slope change × wealth | <0.0001 | 0.995 | -0.006 | 0.006 |
| **Memory** |  |  |  |  |  |
|  | Care for spouse/partner × slope change × wealth | 0.005 | 0.151 | -0.002 | 0.012 |
|  | Not care for spouse/partner × slope change × wealth | 0.001 | 0.619 | -0.004 | 0.007 |
|  | Care for parent/parent-in-law × slope change × wealth | 0.006 | 0.119 | -0.001 | 0.013 |
|  | Not care for parent/parent-in-law × slope change × wealth | 0.002 | 0.445 | -0.004 | 0.008 |
|  | Care for any other × slope change × wealth | 0.000 | 0.957 | -0.008 | 0.007 |
|  | Not care for any other × slope change × wealth | 0.006 | 0.050 | 0.00001 | 0.012 |

**References for Appendix 1**

1. Lee S, Kawachi I, Grodstein F. Does caregiving stress affect cognitive function in older women? J Nerv Ment Dis. 2004;192:51–7. https://doi.org/10.1097/01.nmd.0000106000.02232.30

2. Vitaliano PP, Echeverria D, Yi J, Phillips PEM, Young H, Siegler IC. Psychophysiological Mediators of Caregiver Stress and Differential Cognitive Decline. Psychology and Aging. US: American Psychological Association; 2005;20:402–11. https://doi.org/10.1037/0882-7974.20.3.402

3. Vitaliano PP, Zhang J, Young HM, Caswell LW, Scanlan JM, Echeverria D. Depressed Mood Mediates Decline in Cognitive Processing Speed in Caregivers. Gerontologist. 2009;49:12–22. https://doi.org/10.1093/geront/gnp004

4. Dassel KB, Carr DC, Vitaliano P. Does Caring for a Spouse With Dementia Accelerate Cognitive Decline? Findings From the Health and Retirement Study. Gerontologist. 2017;57:319–28. https://doi.org/10.1093/geront/gnv148

5. Zwar L, König H-H, Hajek A. The impact of different types of informal caregiving on cognitive functioning of older caregivers: Evidence from a longitudinal, population-based study in Germany. Social Science & Medicine. 2018;214:12–9. https://doi.org/10.1016/j.socscimed.2018.07.048

6. Luo Y, Pan X, Zhang Z. Productive activities and cognitive decline among older adults in China: Evidence from the China Health and Retirement Longitudinal Study. Soc Sci Med. 2019;229:96–105. https://doi.org/10.1016/j.socscimed.2018.09.052

7. Yuan J, Grühn D. Time Effects of Informal Caregiving on Cognitive Function and Well-Being: Evidence From ELSA. Gerontologist. 2021;61:341–51. https://doi.org/10.1093/geront/gnaa114

8. García-Castro FJ, Bendayan R, Dobson RJB, Blanca MJ. Cognition in informal caregivers: evidence from an English population study. Aging Ment Health. 2022;26:507–18. https://doi.org/10.1080/13607863.2021.1893270

9. Henning G, Ehrlich U, Gow AJ, Kelle N, Muniz-Terrera G. Longitudinal associations of volunteering, grandparenting, and family care with processing speed: A gender perspective on prosocial activity and cognitive aging in the second half of life. Psychology and Aging. 2023;38:790–807. https://doi.org/10.1037/pag0000780

10. Su Q. Impact of Caregiving on Cognitive Functioning: Evidence From the China Health and Retirement Longitudinal Study. The Journals of Gerontology: Series B. 2023;78:1796–804. https://doi.org/10.1093/geronb/gbad090

11. Bhattacharyya KK, Liu Y, Das Gupta D, Molinari V, Fauth EB. The Healthy Caregiver? A Positive Impact of Informal Caregiving Status on Cognitive Functions Over Time From the Midlife in the United States Study. J Aging Health. SAGE Publications Inc; 2024;36:631–41. https://doi.org/10.1177/08982643231209482

12. Elayoubi J, Nelson ME, Mu CX, Haley WE, Wadley VG, Clay OJ, et al. The role of caregiving in cognitive function and change: The REGARDS study. Psychology and aging. American Psychological Association; 2023;38:712.

13. Guo Y, Zhang Z, Jiang Q. Spousal Caregiving Types and Cognitive Trajectories Among Middle-Aged and Older Adults in China. Res Aging. SAGE Publications Inc; 2025;01640275251317544. https://doi.org/10.1177/01640275251317544

14. Elayoubi J, Haley WE, Walters ME, Roth DL, Howard VJ, Crowe M, et al. Longitudinal impact of transition to caregiving on cognitive functioning: a matched case-control study. J Gerontol B Psychol Sci Soc Sci. 2025;80:gbaf141. https://doi.org/10.1093/geronb/gbaf141
